# Hypercanines: Not just for sabertooths

**DOI:** 10.1002/ar.25510

**Published:** 2024-05-30

**Authors:** Lars Werdelin

**Affiliations:** ^1^ Department of Palaeobiology Swedish Museum of Natural History Stockholm Sweden

**Keywords:** function, hypercanine, mammalia, sabertooth, Synapsida

## Abstract

Hypercanines are here defined as hypertrophied caniniform teeth, that is, canine teeth that are elongated to serve specific functions in different clades of mammals and their synapsid ancestors. This article presents an overview of the occurrence of hypercanines, their growth, and their function across a broad range of clades. Sabertooth felids and felid‐like taxa are found to be unique in having determinate growth (although some Dinocerata may also have this). The most common function of hypercanines among herbivores is found to be sexual display and male–male competition. Three clades of small ruminants have evolved hypercanines that can move within their sockets, although the evolutionary details behind this convergent adaptation have not been worked out.

## 1 INTRODUCTION

This special volume includes articles on selected aspects of the occurrence and evolution of “hypercanines” (actually hypertrophied caniniform teeth, but hypercanines is a more memorable term). These are canine teeth elongated to serve specific functions in different groups of Mammalia and some of their synapsid ancestors. Hypertrophied incisors also occur in many mammals, including most rodents and in some groups where they superficially resemble canines, especially Proboscidea. Hypertrophied incisors will not be discussed here. Hypercanines are mostly but not exclusively from the upper jaw but there are no known mammals with only hypertrophied lower canines.

Arguably the most iconic hypercanines occur in the Felidae and felid‐like predators, that is, “sabertooth cats.” This predatory type has evolved independently in several groups from the Late Paleozoic to the present but is now entirely extinct, leading to a large literature discussing the morphological aspects of the adaptation, as well as its functional and ecological underpinnings. Hypercanines have evolved in many other groups, however, and for a number of other functions. This introductory chapter provides an outline of which groups these are and the various roles hypercanines play in the functional morphology and ecology of different mammals, past and present. These roles are often incompletely known. This applies especially to extinct groups, but in some cases their function in living mammals is largely anecdotal.

There are two types of growth among these among hypercanines: determinate and indeterminate. In the former the roots close at a certain point in development, after which the teeth no longer grow. Hypercanines of this type are mostly found among carnivores but are also present in (at least some) Dinocerata. The second type have ever‐growing teeth, that is, they grow throughout life. Such hypercanines qualify as tusks, for which indeterminate growth is one criterion, and are the more common type of hypercanine, being present in a large number of mammalian clades.

We also include in this review non‐mammaliaform synapsids. These do not have fixed tooth positions, but nevertheless there are among them some taxa with what we may call functional hypercanines (sometimes more than one on either side of the cranium). These include several groups (e.g., gorgonopsians) with determinate grown (but continuous replacement) and one with tusks (dicynodonts).

Because the function of hypercanines is either poorly known in many taxa or they may have multiple functions, this review will be taxonomically based, looking in turn at the different orders of mammals that include hypercanine forms and briefly discussing what we know of their relationships within that order, as well as what is known of their function(s).

## 2 TAXONOMICALLY BASED REVIEW OF HYPERCANINES

### 2.1 Pre‐mammalian Synapsida

Several groups of pre‐mammalian Synapsida included taxa with enlarged caniniform teeth. The majority of these, including Gorgonopsia, Therocephalia, Biarmosuchia, and Dinocephalia, had teeth with determinate growth and continuous tooth replacement, but one, Dicynodontia, had ever‐growing enlarged caniniforms, that is, tusks.

**FIGURE 1:**
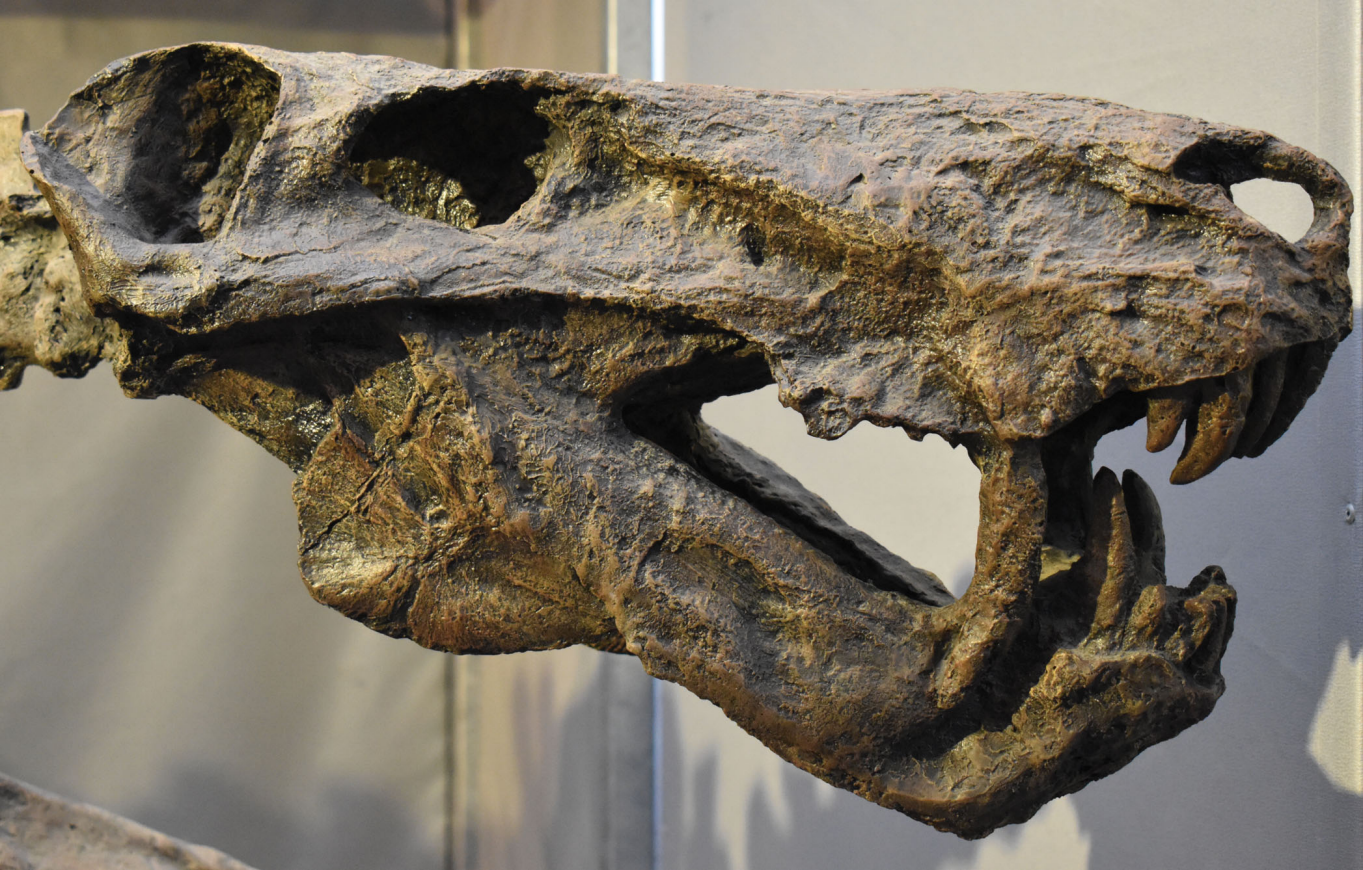
*Inostrancevia alexandri* skull. Photo: Tylwyth Eldar/Wikipedia. Licensed under Creative Commons Attribution‐Share Alike 4.0 International.

Among Gorgonopsia enlarged caniniforms were widespread and sometimes double, presumably as part of solving the problem that tooth replacement can create in which one or both caniniforms are simultaneously replaced. This double caniniform echoes the situation in some machairodont felids in which the deciduous and permanent canines are present simultaneously. In some Gorgonopsia, for example, *Inostrancevia* (Amalitzky, 1922; Figure 1), *Sauroctonus* (Gebauer, 2014) and *Rubidgea* (Kammerer, 2016) the lower canine was also large, but others seemingly had reduced lower caniniforms, just as in machairodont felids, and some even had a lower jaw flange. Reduced lower canines must have evolved as a response to a shift in upper canine function, but how this transition was effectuated has not been explored.

An especially interesting case is the anomodont *Tiarajudens* (Cisneros et al., 2011), which had very long, slender upper canines. This animal was herbivorous, however, and in the cited article the authors suggest that the hypercanines were used as a deterrent to would‐be attackers, much as in some present‐day ungulates (see section 2.4.3).

Most Dicynodontia had tusks, that is, ever‐growing, elongated caniniforms in the upper jaw. In some, the tusks were replaced by bony, tusk‐like protrusions from the maxillae. The function of dicynodont tusks is not entirely clear, but it has been suggested that they may have been sexually dimorphic (Angielczyk et al., 2021) so at least some of their function could have been related to display and reproductive behavior.

In summary, many synapsids had hypercanines in the sense used herein. In some cases these were accompanied by very mammal‐like cheek teeth, and it would be interesting to study the usage of the hypercanines in taxa with continuous tooth replacement to see if insights can be gained into their usage in mammals, where only two tooth generations have had to suffice.

### 2.2 Metatheria

**FIGURE 2:**
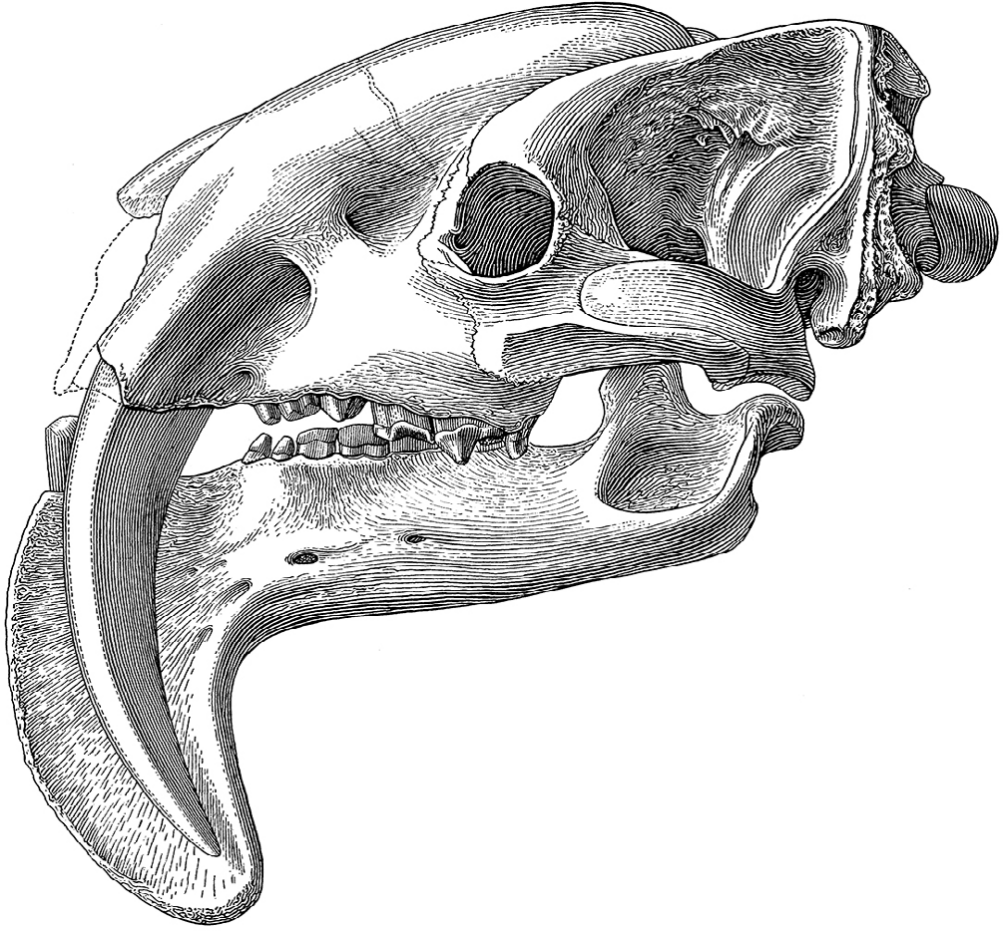
*Thylacosmilus atrox* skull. After Riggs, 1934.

Among Metatheria, one group stands out in the hypercanine stakes: the Thylacosmilidae. This family in the Order Sparassodonta includes three (possibly four) monotypic genera, among which *Thylacosmilus atrox* (Figure 2; Riggs, 1934) is the largest, youngest, and best known. It has been recovered from a number of Late Miocene and Early Pliocene localities in Argentina. The second is *Anachlysictis gracilis* (Suarez et al., 2023), a species from the Middle Miocene of Colombia. The third is *Patagosmilus goini* (Forasiepi & Carlini, 2010) from the Middle Miocene of Argentina. In addition to these, Engelmann et al. (2020) described the species *Eomakhaira molossus* from the Early Oligocene of Chile as a thylacosmilid. However, this has been disputed by Suarez (2022) and Guimarães et al. (2024), who consider it a member of the proborhyaenid stem lineage.

The thylacosmilids are unique among the carnivorous hypercanine taxa in having canines with ever‐growing upper canines, that is, with open roots (e.g., Forasiepi & Carlini, 2010). Thus, by definition their canines are “tusks,” whereas those of placental “sabertooths” are not. The canines of all metatherian carnivores are tusks in this sense, a condition that may related to the monophyodont dentition of the group, and in this respect *Thylacosmilus* is therefore not exceptional.

The functional morphology of thylacosmilids has been debated ever since they first were described. Most studies have taken the view that *Thylacosmilus* (the other genera have not been studied in detail) by analogy with placental sabertooths was a predator (Gaillard et al., 2023; Wroe et al., 2013), although with neck musculature as the main driver of the canine killing bite or stab. On the other hand, Janis et al. (2020) have recently suggested, based on several criteria, that the skull was poorly constructed for such a predatory role. This debate has yet to be resolved.

### 2.3 Eutheria

Many Eutheria have representatives with hypercanines. Some of these are carnivorous but many are herbivorous and use their hypercanines for a variety of purposes. In the following we shall first present carnivorous forms from a diversity of clades, followed by herbivorous forms.

#### 2.3.1 Carnivorous taxa

Oxyaenida, Hyaenodontida, and Carnivora are herein considered to be orders of equal standing because of the uncertainty regarding the phylogenetic relationships between the three groups (Faurby et al., 2021).

##### Oxyaenida

Several sabertooth Oxyaenida have been described. These are united in the Machaeroidinae. This subfamily (or family depending the point of view) is known in western North America from the Wasatchian to Uintan Land Mammal Ages (early and middle Eocene) and in Asia from about the same stratigraphic interval.

**FIGURE 3:**
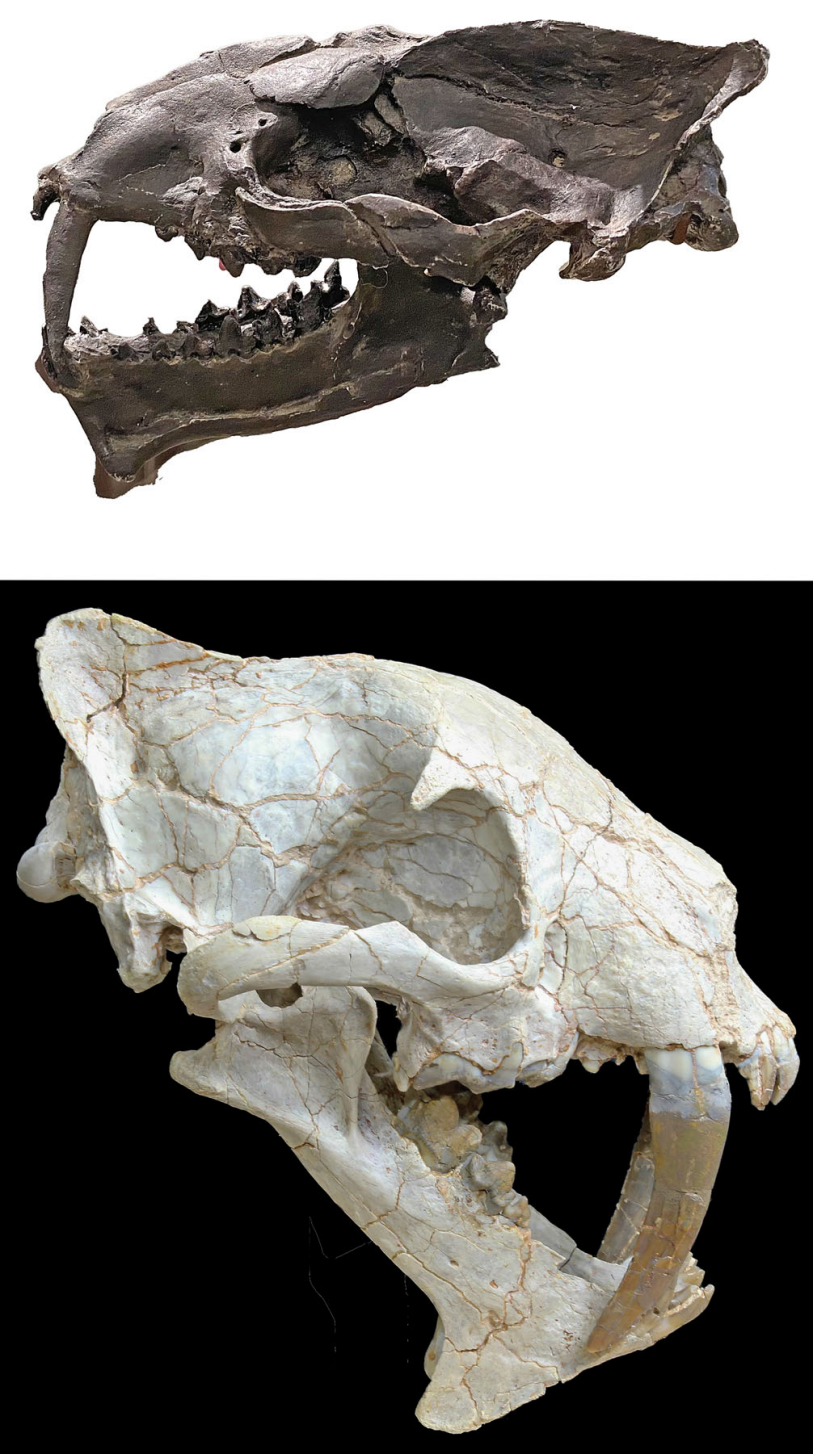
Top: *Machaeroides eothen* skull. Photo: public domain/Wikipedia. Bottom: *Hoplophoneus occidentalis*, skull. Photo: author. (not to scale).

Three genera and four species are known from North America. *Machaeroides*, with two species, *M*. *simpsoni* and *M*. *eothen* (Figure 3 top), *Apataelurus kayi*, and *Diegoaelurus vanvalkenburghae* (Denison, 1938; Zack et al., 2022). A fourth genus, *Malfelis*, represented by *Malfelis badwaterensis* (Stucky & Hardy, 2007), assigned to Machaeroidinae is based on a juvenile specimen with deciduous dentition. The deciduous canines, studied through CT‐scans, appear flattened but the interpretation of this genus as a sabertooth must be considered tentative. If this interpretation is correct, it is by far the largest machaeroidine.

Of the genera that clearly are sabertoothed, *Machaeroides* is older and more primitive, with *M*. *simpsoni* showing only very modest adaptations to the sabertooth gestalt, and *Machaeroides eothen* somewhat more derived. The genera *Apataelurus* and *Diegoaelurus* are of similar stage of evolution, slightly more derived than *M*. *eothen*, but *Diegoaelurus* in particular is considerably larger.

Machaeroidinae are also known from Asia but from very poor material. *Apataelurus pishigouensis* is known by a single specimen with p4‐m1 from Henan, China and *Isphanatherium ferganensis* from an isolated M2 from the Fergana Basin, Central Asia. Both are from the Irdinmanhan Land Mammal Age (roughly late lower Eocene–late middle Eocene).

All machaeroidines are poorly known although the record of the group is improving. Zack (2019) recently assigned a partial skeleton to the Machaeroidinae, hitherto the only known postcrania of the order. The functional analysis of this skeleton suggests scansorial or arboreal habits. The Machaeroidinae may be important in providing a parallel case of rapid evolution from primitive to derived sabertooth morphology for comparison with instances in Carnivora. In addition, the presence of machaeroidine sabertooths begs the question of why this adaptation is not known in Hyaenodonta.

##### Hyaenodonta

As noted, sabertooth adaptations have as yet not been described in Hyaenodonta. Why this should be is unclear.

##### Carnivora

The sabertooth adaptation evolved at least twice and possibly as many as four times in Carnivora. The earliest Carnivoran (or Carnivoramorphan depending on which phylogeny is accepted) sabertooths were the Nimravidae, a family almost exclusively comprising sabertooth forms. The earliest Nimravidae are from China, where several finds of middle‐late Eocene nimravids have been made, including the cranium of *Maofelis cantonensis* (Averianov et al., 2016). Nimravids appear in the late Eocene in both Europe and North America and diversify on both continents from the late Eocene to the middle of the Oligocene and then disappear from both continents by the late Oligocene (Barrett, 2016; Peigné, 2003).

Barrett (2021) presents an interesting and plausible view of the Paleogene evolution of Nimravidae. He finds two distinct clades, Hoplophoninae (Figure 3 bottom) and Nimravinae. The first of these rapidly evolved advanced sabertooth forms that became extinct prior to 30 Ma. The second lineage includes more Felinae‐like forms (although still clearly sabertooth in character, comparable to *Metailurus* and *Dinofelis* among Felidae). This lineage survives into the late Oligocene.

This is not the end of the nimravid story, however. After a gap of ca. 5 million years non‐Felidae sabertooths reappear, this time in the Early Miocene of Africa, where two genera are recognized, *Ginsburgsmilus* and *Afrosmilus* (Werdelin, 2021). These and later Eurasian and North American species have been generally referred to the family Barbourofelidae (Schultz et al., 1970). More recently, however, Barbourofelidae has been viewed as a subfamily within Nimravidae (Barrett et al., 2021), a view that has received support from recent finds in China (Wang et al., 2020). There are unresolved problems with this scenario, however, quite apart from the temporal gap between nimravids and barbourofelids. The two African Miocene genera are very primitive compared to Miocene barbourofelids from Eurasia and North America as well as from the Nimravidae of the Eocene and Oligocene. *Ginsburgsmilus*, in particular, is very primitive for a sabertooth, with only the moderately flattened canine hinting at this adaptation. *Afrosmilus* is more derived and links *Ginsburgsmilus* to the sabertooth gestalt. These genera require either that sabertooths “started over” in the Early Miocene of Africa or these taxa had nothing to do with the subsequent evolution of Barbourofelidae. The second option increases the “cat gap” between Nimravidae and Barbourofelidae by an additional 2–3 million years. Regardless of which scenario and set of relationships is correct, the sabertooth adaptation can be seen to have evolved twice: once at the origin of Nimravidae and once either at the origin of Barbourofelinae in Africa or in the *Ginsburgsmilus*–*Afrosmilus* lineage separately from Barbourofelidae.

The first sabertooth Felidae (Machairodontinae) appear in the Early Miocene with the European *Pseudaelurus quadridentatus*. Sabertooths are poorly known until the late middle Miocene, when *Miomachairodus pseudaeluroides* (Schmidt‐Kittler, 1976; Viranta & Werdelin, 2003) appears, followed by *Machairodus* spp. Reading the fossil record literally the lineage splits into the two tribes Homotherini and Smilodontini in the Late Miocene. However, recent studies of ancient mtDNA (Paijmans et al., 2017; Westbury et al., 2021) suggest that the two tribes diverged much earlier, in the Early Miocene (around 18–20 Ma). This, in turn, indicates a split within *P*. *quadridentatus* at an early stage of its evolution. In consequence, since *P*. *quadridentatus* has only the slightest indications of sabertooth morphology (just like *Ginsburgsmilus*) it appears likely that the sabertooth gestalt evolved independently in Homotherini and Smilodontini. The reasons for the parallel occurrence of two lineages that seem to have coexisted geographically at least since the early Late Miocene (Siliceo et al., 2014) can for now only be speculated on, but may relate to habitat use if the distinctive postcranial adaptations of *Homotherium* and *Smilodon* can be followed back in the fossil record.

In summary, the sabertooth gestalt appears to have evolved four times among Carnivoramorpha, with all lineages except Nimravinae ending with rather extreme forms such as *Hoplophoneus*, *Eusmilus*, *Barbourofelis*, *Homotherium*, and *Smilodon*. This is congruent with the concept of the evolutionary ratchet (Holliday & Steppan, 2004). If a direct link between Nimravinae and *Ginsburgsmilus/Afrosmilus* can be found this would provide further evidence that the other sabertooth lineages had reached their “point of no return” prior to extinction.

##### Odobenidae

A final Carnivoran taxon with hypercanines is the walruses. The extant walrus has the longest canines of any living species (and possibly all fossil species as well). This iconic feature is relatively new in walrus evolution, however, only having appeared in the Late Miocene with the subfamilies Dusignathinae and Odobeninae. This likely signals a shift in feeding strategy compared to earlier taxa such as *Titanotaria*, which lacked tusks. It has been thought that the tusks were used for pulling up mollusks from the sea floor. However, recent studies of dental wear indicate that the tusks were dragged along the sea floor, whereas the snout was used for foraging. Instead, the tusks appear to primarily be used for intraspecific combat (Fay, 1982; Ray et al., 2006).

### 2.4 Non‐carnivorous taxa

A large number of non‐carnivorous mammalian taxa at the ordinal level or below have evolved hypercanines. The function of these teeth varies from group to group, as does our knowledge of them. The following exposé will begin with extinct taxa and continue with the extant taxa, including a discussion of their fossil representatives, if relevant.

#### 2.4.1 Dinocerata

**FIGURE 4:**
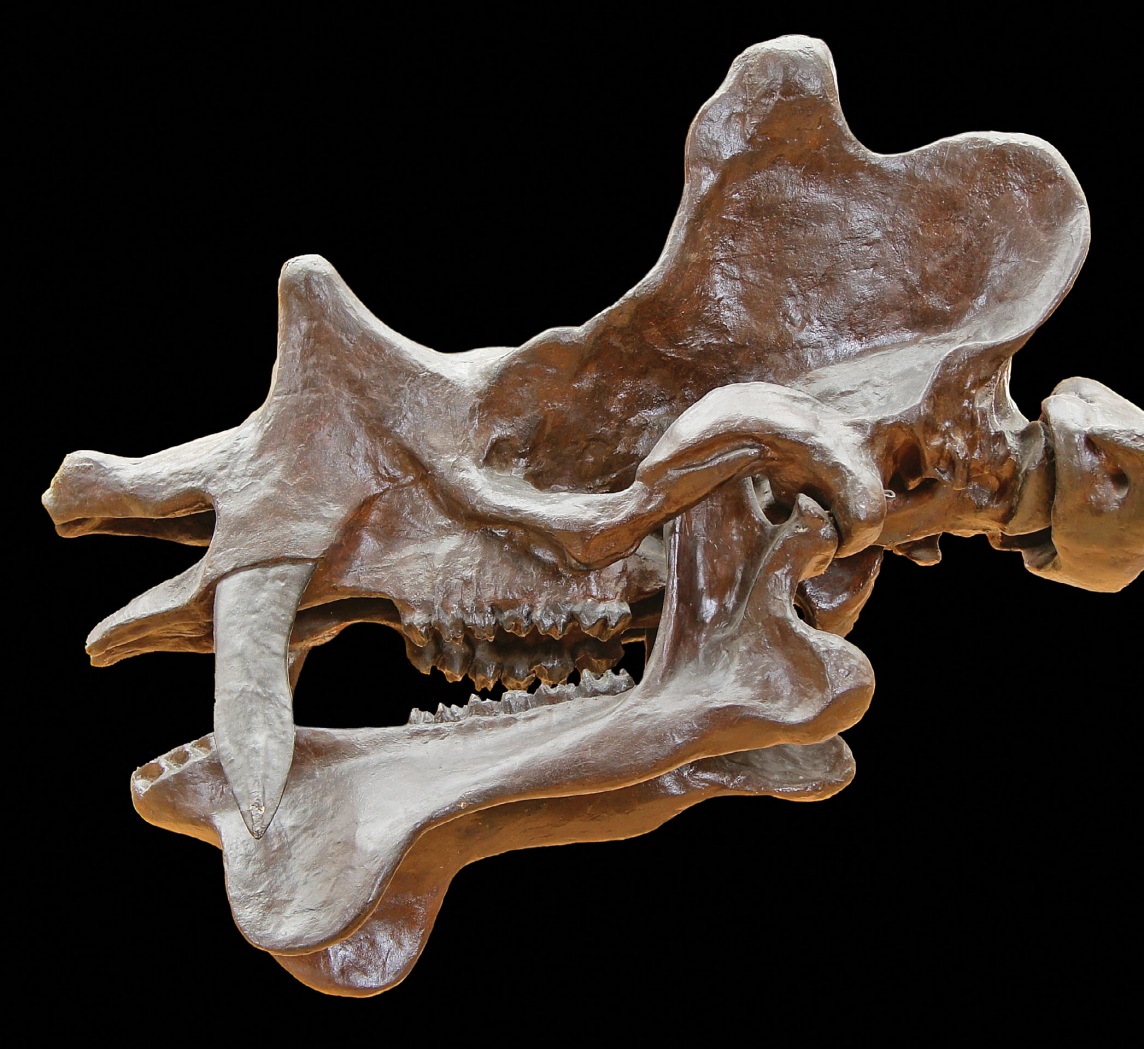
*Dinoceras mirabile*, skull. Photo: public domain/Wikipedia.

The Dinocerata (or uintatheres) is a group of medium‐ to large‐sized, herbivorous, mainly graviportal ungulates found in Paleocene and Eocene sediments in North America and eastern Asia. They are unique in a number of features, such as the lanceolate shape of the canine tips of at least some representatives (such as *Uintatherium*; Figure 4), their largely concave rather than convex cranial structure, and their bony, horn‐like protuberances on the cranium. Like many sabertooths they have an anterior mandibular flange that protects the canine.

The canine roots appear to be open in young individuals but close later in life, at least in forms with lanceolate canine tips. They are quite flattened, more so than in, for example, “sabertoothed” deer (see below) and have a curvature similar to that of sabertoothed predators. Marsh (1886) suggested that Dinocerata (or at least *Uintatherium*) were sexually dimorphic, with females having smaller and more slender canines (among other features). This suggestion has been followed by subsequent authors (Flora, 2021; Turnbull, 2002; Wheeler, 1961) and indicates that display may have been part of the function of the canines, together with defense.

#### 2.4.2 Astrapotheria

**FIGURE 5:**
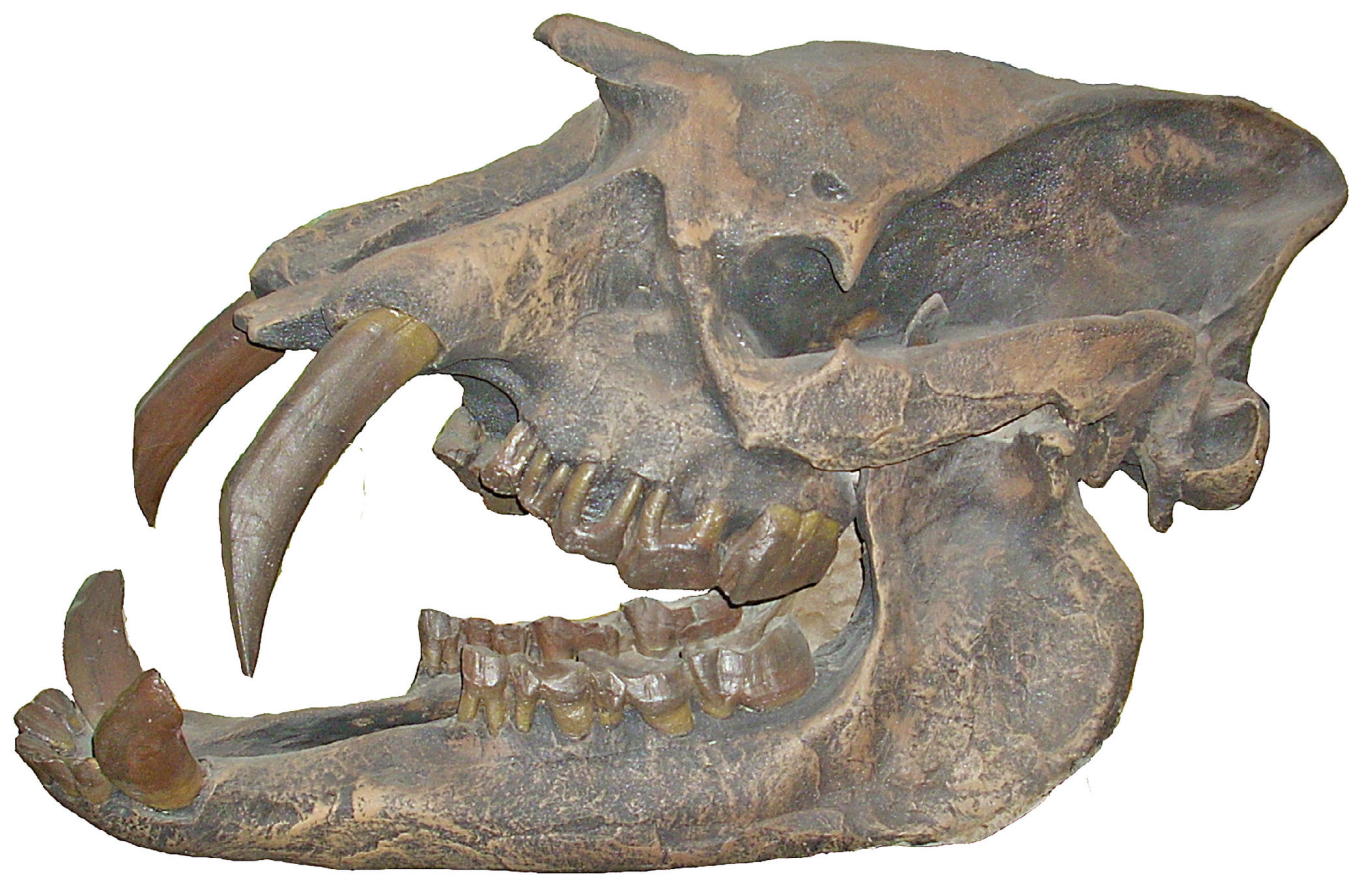
*Astrapotherium* sp. skull. Photo: Gastón Cuello/Wikipedia; modified by removing background. Licensed under CC BY‐SA 3.0.

Astrapotheres constitute an extinct order of South America ungulates. They are known from Paleocene to Middle Miocene levels, mainly but not exclusively in southern South America. Their relationships primarily lie with other South American ungulates. Recent proteomic studies suggest that some groups of South American ungulates, such as *Macrauchenia* (Litopterna) and *Toxodon* (Notoungulata), were related to the panPerissodactyla (Laurasiatheria) (Welker et al., 2015; Westbury et al., 2017). Such studies have not been carried out on Astrapotheria and their potential relationship to other clades is poorly known. The best known astrapothere is *Astrapotherium* (Figure 5), which was a large‐sized, graviportal form. It had relatively weak limbs and has been suggested to be semiaquatic—an early South American analog to hippos or tapirs. They may also have had a small proboscis, similar to that of tapirs. *Astrapotherium* had enlarged upper and lower canine tusks that occluded for self‐honing. Their function is not clear, but the honing occlusion suggests that they may have been defensive structures as well as being used for intraspecific combat (similar to hippos).

#### 2.4.3 Artiodactyla

A number of artiodactyl clades have elongated canines (Cabrera & Stankowich, 2018). These canines show a series of interesting, parallel adaptations in the different clades, though these have not been adequately studied from an evolutionary standpoint.

The Tragulidae (chevrotains or mouse deer) is the first of four taxa of artiodactyl ruminants to have evolved hypercanines, apparently independently. Despite the trivial name they are not deer. According to current thinking, Tragulidae is the extant sister taxon to Pecora, the group that includes all other ruminants, including true deer. Tragulidae are known as far back as the late Eocene (Mennecart et al., 2021; Metais et al., 2007) although it is not known whether they had hypercanines at this early date. Enlarged canines were, however, certainly present in *Dorcatherium* from the Late Miocene of Europe (Hartung & Böhme, 2022). The canines are sexually dimorphic in extant chevrotains, with males having larger canines and females smaller ones, although this is apparently variable between species. I have found no statement that they could move in the alveoli, but the large alveoli relative to canine size (Meijaard & Groves, 2004) suggest that this is at least possible to some degree. Intra‐specific combat and possibly defense are the likely functions of the hypercanines.

**FIGURE 6:**
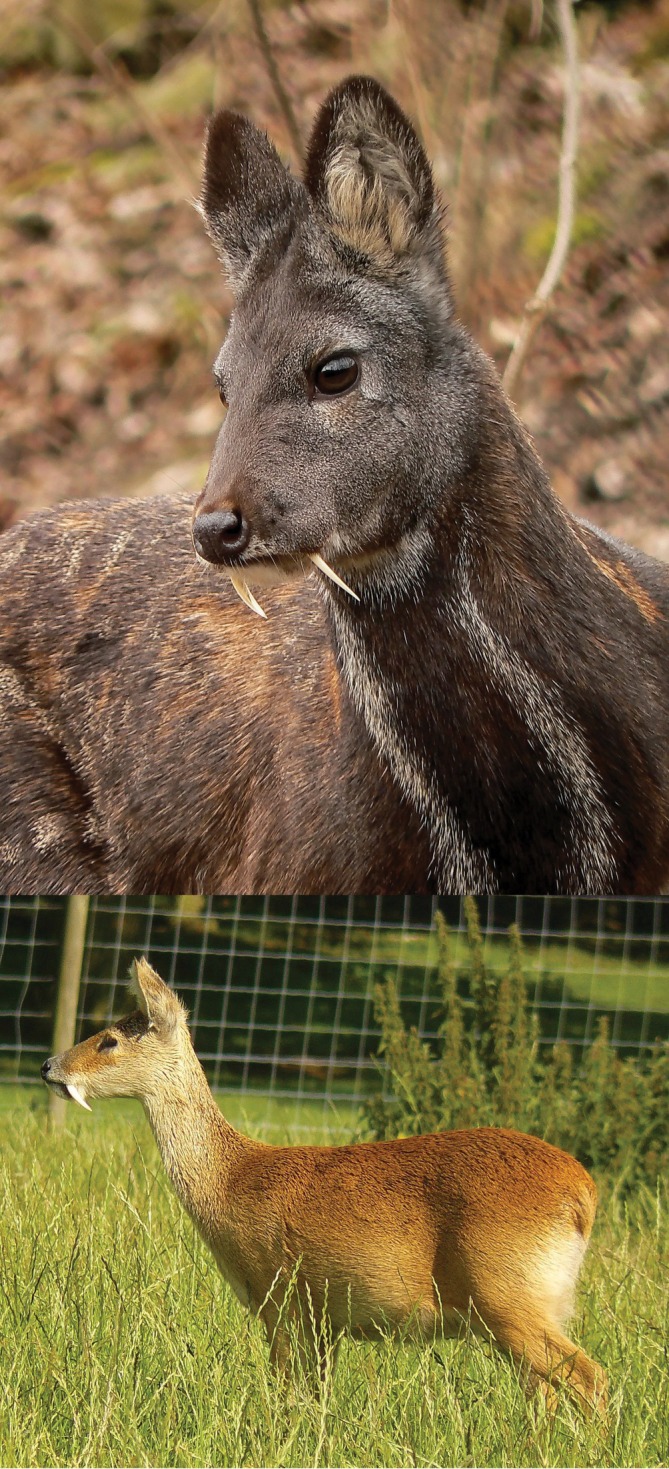
Top: *Moschus moschiferus* (musk deer). Photo: Nikolai Usik/Wikipedia. Licensed under CC BY‐SA 3.0. Bottom: *Hydropotes inermis* (water deer). Photo: public domain/Wikipedia. Both are images of zoo animals.

Moschidae (musk deer), like chevrotains, are not actually deer, but the sister taxon to Bovidae, with these two in turn being sister to Cervidae. Musk deer are known from the Oligocene to present. They were common in the Miocene of North America and Europe, with genera such as *Blastomeryx* and *Micromeryx* (Janis et al., 1998; Rössner & Heissig, 1999), although it should be noted that the relationship of Blastomerycinae to Moschidae is debated. The Late Miocene saw a major extinction of Moschidae and today the family is represented by the single genus *Moschus* (Figure 6 top) with about seven species in Asia. The Miocene extinct forms already had the hypercanines seen in the living species. These, which can swivel antero‐posteriorly in the sockets to enable feeding, are only present in males. Because of this, like Tragulidae the function of the hypercanines in musk deer seems to primarily be interspecific combat or display, but they may also be involved in defense.

Two taxa of true deer have hypercanines, the water deer (*Hydropotes*) and the muntjacs (*Muntiacus* and *Elaphodus*). These are quite distantly related, belonging in separate subfamilies (Capreolinae and Cervinae, respectively).

*Hydropotes* (Figure 6 bottom) has no fossil record so beyond phylogeny nothing is known about its antecedents. The modern species, *H*. *inermis*, is found in China and the Korean peninsula., with extralimital records being recorded from eastern Russia. An invasive (introduced) population occurs in Great Britain. *Hydropotes* is unique among Cervidae in lacking antlers. The canines are set loosely in the sockets so that facial musculature can be used to swivel them backward for feeding and forward for defense and intraspecific fighting. Aitchison (1946) states that the canines are primarily used for intraspecific combat.

The Tribe Muntiacini consists of two genera, *Muntiacus* (barking deer) with 12 currently recognized species, and *Elaphodus* (tufted deer) with the single species *E*. *cephalophus*. Both genera are native to southeast Asia although like the water deer there is an introduced population of *Muntiacus reevesi* distributed across Great Britain. Muntjacs are known in the fossil record from at least the Late Miocene of the Qinghai‐Tibetan Plateau (Dong, 2007). The upper canines have the same specialized adaptations that are present in *Hydropotes*. According to Aitchison (1946) the tusks of muntjacs are secondary weapons in combat when the opponent has come too close for antler fighting.

The remarkable convergence of the hinged canine teeth has its obvious functional correlate in the need for these animals not only to feed on their vegetation of choice (especially relevant for grazers) but also to ruminate. Both of these functions require transverse jaw movement, and the hinged canines may be an adaptation to allow for this, as discussed in Aitchison (1946) and Avedik et al. (2023). Given the distant relationships between the artiodactyls with hinged canines it would be expected that the mechanisms should differ in detail, but this has apparently not been studied, possibly because until recently the relevant taxa have been considered closely related.

**FIGURE 7:**
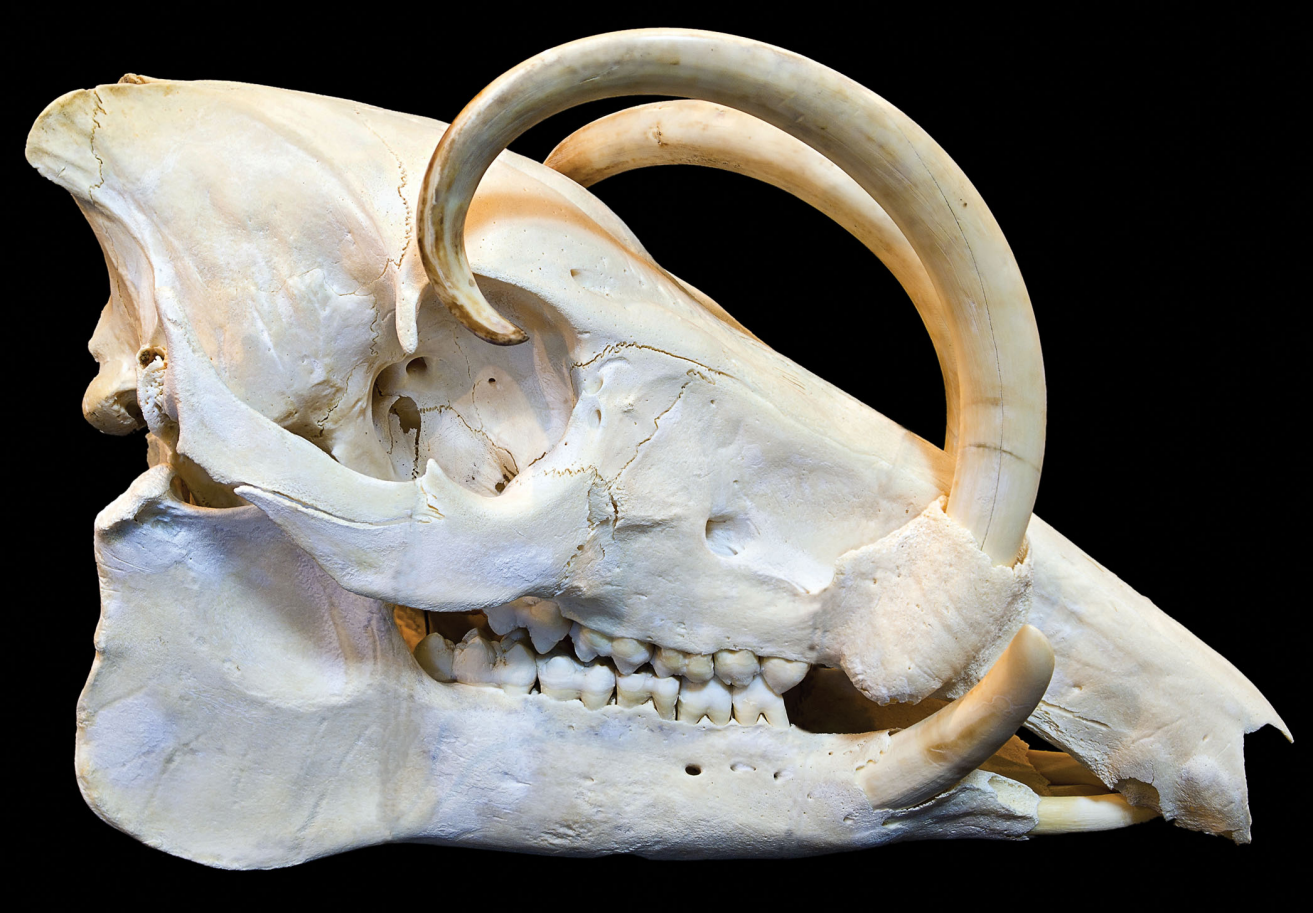
*Babirousa celebensis* skull. Photo: Didier Descouens/Wikipedia. Licensed under Creative Commons Attribution‐Share Alike 4.0 International.

Suidae includes many taxa with canine tusks that vary in structure and function from species to species. In, for example, warthogs (*Phacochoerus* spp.) and wild boar (*Sus scrofa*) they are very large and sharp. In most suids that do have tusks they make formidable weapons, chiefly for defense. In addition to this, the Suidae include a species with what may be the most extreme development of canine tusks in Mammalia. This is the babirusa (*Babirousa* spp.; Figure 7). In this genus the upper canines of the male grow vertically from the maxilla, penetrate the flesh of the snout and curve over the face. These canines are clearly used for display since they are poorly placed for combat. In other species, such as the red river hog (*Potamocherus porcus*), the tusks are comparatively small, but still functional weapons in combat.

Hippopotamidae appear in the Miocene, with the Middle Miocene *Kenyapotamus* as a possible ancestral taxon. Most fossil hippos appear to have had tusks of relatively great size although there is a clear size difference between the extant species *Hippopotamus amphibius* (common hippo) and *Choeropsis liberiensis* (pygmy hippo) with the latter having absolutely and relatively smaller canines. The canines are formidable weapons in both defense and intra‐specific combat.

## 3 DISCUSSION

“Hypercanines” is the term here used for hypertrophied caniniform teeth, that is, canines or caniniforms that have enlarged beyond the “standard” canine length for a specific group of animals. Although to some extent subjective, this definition serves adequately in practice, acknowledging that there may be some borderline cases, however.

Hypercanines are present in a broad range of mammals and pre‐mammalian synapsids. They have a range of functions that include food procurement, defense, and reproductive behavior.

Food procurement is exclusively present in predatory taxa, from gorgonopsians to sabertooths. There is no clear evidence that hypercanines functioned in food procurement in any herbivorous mammals despite suggestions to that effect for a number of taxa (e.g., Dinocerata, Odobenidae). Hypercanines as carnivore killing devices are present as three types. The earliest is in taxa with continuous tooth replacement. In these animals hypercanines are generally upper caniniforms, but rarely also lower caniniforms. Tooth replacement leads to there sometimes being zero or two hypercanines in a given side of the maxilla.

Mammalian sabertooth predators all have hypercanines associated with relatively reduced lower canines. The hypercanines in these groups all have closed roots and therefore finite growth. The sole exception to this is the South American Thylacosmilidae which have open roots and therefore indeterminate growth. On the other hand, there is ongoing debate regarding whether these animals (or at least the terminal form, *Thylacosmilus atrox*) was truly a predator, for example, Janis et al. (2020) and references therein. There is no evidence for sexual dimorphism in any of the above animals.

The defense, interspecific combat, and display functions for hypercanines are difficult to distinguish and may in practice be the same. After all, teeth that can be used to fight other taxa could also be used for fighting with conspecifics or for display, as long as they are externally visible. Thus, despite outward appearance the hypercanines of such disparate animals as dicynodonts, musk deer, and walruses likely had the same function (or functions). The majority of these hypercanines had open roots and indeterminate growth, qualifying them to be identified as tusks.

Most remarkable in this overview of hypercanines is the convergence between three groups of distantly related artiodactyls on a similar solution to the defense/intraspecific combat/display function(s). The solution that involves canines that can swivel in their alveoli controlled by facial musculature deserves renewed, detailed study from an evolutionary perspective.

## AUTHOR CONTRIBUTIONS

**Lars Werdelin:** Conceptualization; writing – original draft; writing – review and editing.
